# Verification of the Nutritional and Dietary Factors Associated with Skeletal Muscle Index in Japanese Patients with Nonalcoholic Fatty Liver Disease

**DOI:** 10.1155/2020/3576974

**Published:** 2020-07-09

**Authors:** Takashi Himoto, Keiko Miyatake, Takashi Maeba, Tsutomu Masaki

**Affiliations:** ^1^Department of Medical Technology, Kagawa Prefectural University of Health Sciences, 281-1, Hara, Mure-Cho, Takamatsu, Kagawa 761-0123, Japan; ^2^Department of Nutrition Management, Ritsurin Hospital, 3-5-9, Ritsurin-Cho, Takamatsu, Kagawa 760-0073, Japan; ^3^Department of Surgery, Ritsurin Hospital, 3-5-9, Ritsurin-Cho, Takamatsu, Kagawa 760-0073, Japan; ^4^Department of Gastroenterology and Neurology, Kagawa University School of Medicine, 1750-1, Ikenobe, Miki-Cho, Kagawa 761-0793, Japan

## Abstract

We sought to identify the frequencies of presarcopenia, sarcopenia, and sarcopenic obesity in patients with nonalcoholic fatty liver disease (NAFLD) and to cross-sectionally determine the nutritional and dietary factors associated with loss of skeletal muscle mass in such patients. Dietary and body component changes produced by a diet intervention were longitudinally investigated. Forty-six NAFLD patients (24 males and 22 females) were enrolled. A second diet treatment was performed at 6 months after entry in 19 of the enrolled patients (6 males and 13 females). Body compositions and dietary nutrients at six months later were compared with those at entry. Three of the 24 (13%) males and four of the 22 (18%) females fulfilled the criteria for presarcopenia and one (5%) female NAFLD patient was in the criteria for sarcopenia at baseline. None of the patients were in the criteria for sarcopenic obesity. The factors associated with skeletal muscle index in the males were body mass index (BMI), insulin-like growth factor-1, total energy intake, and lipid intake, but only BMI and bone mineral density in females at baseline. The diet intervention decreased the skeletal muscle mass in the 6 males by decreasing the total energy intake via lower protein and lipid intakes and improved their liver dysfunction. In the 13 females, a decrease in total energy intake via lower carbohydrate and lipid intake did not change the skeletal muscle mass. These results suggest that loss of skeletal muscle mass is frequently observed in nonobese NAFLD patients and that the frequency of sarcopenic obesity seems to be rare in NAFLD patients. The nutritional and dietary factors that regulate loss of skeletal muscle mass were distinct between our male and female NAFLD patients. Thus, the skeletal muscle mass of such patients as well as their body weight and liver function should be monitored during diet interventions.

## 1. Introduction

Sarcopenia, a concept proposed by Rosenberg [1] in 1989, characterized by loss of skeletal muscle mass and low muscle strength, is widely recognized as a primary factor responsible for frailty [2] and is regulated by muscle mass, muscle strength, and physical performance [3]. The clinical outcomes of sarcopenia in Asian countries appears to be more serious than those in Western countries, because in Asian countries, the aging of the population is in rapid progress and the proportion of older individuals continues to increase. The physical status, ethnicity, and lifestyle in Asian countries differ in many ways from those in Western countries. Therefore, original diagnostic criteria for sarcopenia that are appropriate for Asian people were required [4]. However, outcome-based data were not available in Asian Working Group for Sarcopenia (AWGS). Therefore, the working group aimed to standardize the cutoff values for evaluating sarcopenia in Asian people.

Sarcopenia is largely classified into two categories: primary sarcopenia and secondary sarcopenia [3]. Primary sarcopenia is called “age-related sarcopenia.” Secondary sarcopenia includes activity-related sarcopenia, disease-related sarcopenia (e.g., type 2 diabetes mellitus (T2DM), liver cirrhosis, osteoporosis, and varieties of malignant diseases), and nutrition-related sarcopenia such as malnutrition [3]. Sarcopenia is associated with not only numerous hormonal factors, including insulin-like growth factor-1 (IGF-1) [5], insulin [6], testosterone [7], and oxytocin [8] but also nutritional factors such as trace elements [9, 10], vitamin D, protein, leucine [11], polyunsaturated fatty acid (PUFA) [12], and body mass index (BMI) [13]. Inflammatory cytokines and myokines, including interleukin-6 (IL-6) and myostatin, are also thought to affect the skeletal muscle mass [14].

In the field of liver disease, many investigators primarily have focused on patients with liver cirrhosis as a target of the researches on sarcopenia [15]. The quality of life among cirrhotic patients associated with sarcopenia is severely impaired, and the prognosis of these patients is quite unfavorable. Several studies following the report by Hong and colleagues [16] in 2014 have indicated that sarcopenia was present even in patients with nonalcoholic fatty liver disease (NAFLD), implying that NAFLD might be one of the risk factors for sarcopenia [17–21].

NAFLD, characterized by a spectrum ranging from simple steatosis to steatohepatitis without a habit of excessive alcohol consumption, is one of the most prevalent chronic liver diseases worldwide [22]. NAFLD is frequently associated with metabolic abnormalities, including obesity, impaired glucose tolerance, and dyslipidemia [23]. Accordingly, diet and/or exercise treatment should be recommended as a first choice of therapies for NAFLD patients [24]. However, the effects of diet treatment for NAFLD patients on the patients' skeletal muscle mass have not been fully established.

“Sarcopenic obesity,” a comorbid disorder of sarcopenia and obesity [25], is estimated to be present in approximately 3%–20% of older populations [26]. The prognosis of patients with sarcopenic obesity appears to be much more unfavorable than that of patients with obesity or sarcopenia alone [27]. We have speculated that some patients with sarcopenic obesity are hidden among the NAFLD patients who are obese, but there are few published data regarding sarcopenic obesity in patients with NAFLD.

The primary purposes of this study were to identify the frequencies of sarcopenia and sarcopenic obesity in Japanese patients with NAFLD and to determine which factors are the most responsible for loss of skeletal muscle mass in these patients. We also sought to establish whether a diet treatment for patients with NAFLD would affect their skeletal muscle mass as well as the body fat mass and liver function.

## 2. Materials and Methods

### 2.1. Study Design

This study was designed by a cross-sectional and longitudinal trial. Nutritional and dietary factors were analyzed in each participant at baseline. Dietary intervention by a dietitian was also performed without any medications. In addition, the participants were urged to perform the physical activities, including aerobics and resistance exercise training, for 30 minutes three times a week. Changes in body compositions and nutrients were also analyzed 6 months later among the participants who could follow up.

### 2.2. Study Population

We enrolled 46 patients with NAFLD (24 males and 22 females) from the outpatients of Ritsurin Hospital (Takamatsu, Japan) treated between 2016 and 2018. None of the enrolled NAFLD patients had a habit of excessive daily alcohol consumption, and all patients showed radiological findings by abdominal ultrasound and/or computed tomography (CT) test that are compatible with fatty liver. NAFLD patients who took medication for hypertension, dyslipidemia, and/or T2DM were excluded in this study.

The study protocol complied with all of the provisions of the Declaration of Helsinki. The design of this study was approved by the Ethical Committee of Ritsurin Hospital, and informed consent was obtained from each patient before his or her entry in this study.

### 2.3. Laboratory Assessments

Varieties of clinical parameters in the 46 patients were measured to verify the relation to sarcopenia at baseline. Fasting blood samples were taken in the morning for the measurements of plasma glucose and serum immunoreactive insulin (IRI), alanine aminotransferase (ALT), total cholesterol (T-Cho), LDL-cholesterol (LDL-C), triglyceride (TG), and free testosterone levels. The plasma glucose and serum IRI, AST, ALT, T-Cho, and TG levels , HbA1c, and peripheral platelet counts were measured using standard laboratory techniques. The serum free testosterone levels were determined by an immune radio metric assay (IRMA). Insulin resistance was evaluated based on the homeostasis model for the assessment of insulin resistance (HOMA-IR) value using the following equation: HOMA-IR value = fasting insulin (*μ*U/ml) × fasting glucose (mg/dl)/405. Insulin resistance was defined as a HOMA-IR value exceeding 2.5. The measurement of serum IGF-1 concentrations performed using a commercially available kit (TFB Inc, Tokyo, Japan). The assay of IGF-1 was canceled from the enrolled patients who were younger than 40 years old because aging affects the release of the hormone. The serum zinc levels and 25-OH vitamin D_3_ levels were determined by the atomic absorption method and a double-antibody radioimmunoassay, respectively. The clinical diagnosis of zinc deficiency (less than 60 *μ*g/dl) and that of 25-OH vitamin D_3_ deficiency (less than 20 ng/ml) were based on the previous reports [28, 29], respectively. Plasma branched-chain amino acids (BCAA: valine, leucine, and isoleucine) and tyrosine levels were determined by the high-performance liquid chromatography (HPLC) method, and the BCAA to tyrosine ratio (BTR) was calculated. The patients' bone mineral density (BMD) was determined at the lumbar spine (L2-L4) using a DEXA system (GE Health Care, Madison, WI). Osteoporosis was defined as a BMD value less than 70% of the young adult mean (YAM).

Body mass index (BMI) was estimated as a hallmark of obesity. Obesity was defined as a BMI over 25.0 kg/m^2^, because the reported proportion of the Japanese population with a BMI higher than 30 kg/m^2^ is 2%-3% [30], whereas the proportion of obesity in Western countries ranges from 10% to 20% [31]. Dyslipidemia was defined as a serum LDL-C level exceeding 140 mg/dl and/or TG level exceeding 150 mg/dl [32]. The diagnosis of T2DM was made on the basis of the exclusion of insulin depletion and a fasting plasma glucose level exceeding 126 mg/dl, an oral glucose tolerance test-2 hr plasma glucose level ≥200 mg/dl, a casual plasma glucose level ≥200 mg/dl, and/or a HbA1c level ≥6.5% [33]. Hypertension was designated as blood pressure exceeding 140/90 mmHg [34]. The severity of hepatic fibrosis was estimated by calculating the FIB-4 index as follows: age (years) × AST (IU/L)/platelet count (×10^9^/L)×$\sqrt{\text{ALT}}$ (IU/L) [35].

### 2.4. Criteria for Sarcopenia

For the analysis of body compositions, the skeletal muscle mass of each patient's entire body and that of the limbs, the amount of body fat, and the body fat percentage were measured by a direct segmental multifrequency bioelectric impedance analysis (BIA) using an InBody 470 system (InBody Japan, Osaka, Japan).

Sarcopenia was defined as both loss of skeletal muscle mass and a decline in hand grip strength on the basis of the Sarcopenia Assessment Criteria issued by the Japan Society of Hepatology [36]. We calculated each patient's skeletal muscle index (SMI) [37], an indicator for loss of skeletal muscle mass by using the following formula: SMI = the appendicular skeletal muscle mass (kg)/height (m)^2^. We set 7.0 kg/m^2^ or less for the males and 5.7 kg/m^2^ or less for the females as the values that indicate loss of skeletal muscle mass.

Hand grip strength was measured in each patient in a standing position using a hand dynamometer (TKK5401, Takei, Niigata, Japan). We designated 26 kg or less for the males and 18 kg or less for the females as decreased muscle strength.

Sarcopenic obesity was defined in this study as both sarcopenia and obesity, because a precise definition of sarcopenic obesity has not been established. Presarcopenia was defined herein as loss of skeletal muscle mass alone. The definition of hidden obesity was based on a BMI less than 25 and a body fat percentage exceeding 25% in the males and 30% in the females, respectively.

### 2.5. Dietary Assessments

The patients' dietary intake was assessed with the use of three day diet diaries at baseline and 6 month later. The total energy, protein, leucine, carbohydrate, and lipid intakes were estimated in each patient by the Standard Tables of Food Composition in Japan-2015 [38].

### 2.6. Statistical Analyses

Data are represented as the mean ± standard deviation (SD). The Mann–Whitney *U* test was applied for two groups. Fisher's exact probability test was used to compare the differences in frequencies. The relationships among quantitative variables were analyzed by Spearman's correlation coefficient. The paired *t* test was used to compare the valuables at baseline and 6 months later. *p* values less than 0.05 were considered significant.

## 3. Results

### 3.1. Patients' Characteristics

The clinical characteristics of the 46 NAFLD patients (24 males, 22 females) enrolled in this study at baseline are shown in Table 1. The age was lower in male patients than in female patients but not significant. Concurrent obesity (BMI ≥25) was present in 15 (63%) of the 24 male and 11 (50%) of the 22 female patients, respectively. There was no significant difference in BMI between the groups.

The values of HOMA-IR ranged from 0.82 to 5.2 among ten male and nine female patients. Four of these 10 (40%) males and four of the nine (44.4%) females met the category for insulin resistance, respectively. The values of HOMA-IR were almost equal between the males and the females.

Zinc deficiency was identified in three of the 23 males (13.0%) and four of the 22 females (18.2%), respectively. The frequency of vitamin D_3_ deficiency was higher in female than in male patients (81.8% vs. 70.0%, *p* > 0.05).

The FIB-4 index ranged from 0.4 to 2.25 in the males and from 0.64 to 3.49 in the females, respectively. The FIB-4 index in two of the females exceeded 2.67 [39], which corresponded to liver cirrhosis. The BMD analysis revealed that another female patient had osteoporosis.

The frequencies of concurrent life style-related diseases, including T2DM, dyslipidemia, and hypertension, were approximately equivalent between the males and females.

### 3.2. Frequencies of Presarcopenia, Sarcopenia, and Sarcopenic Obesity

As shown in Table 2, three of the 24 (13%) male NAFLD patients fulfilled the criteria for presarcopenia, and none of the males met the criteria for sarcopenia or sarcopenic obesity at entry. Among the 22 female NAFLD patients, four (18%) met the criteria for presarcopenia and one (5%) met the criteria for sarcopenia, respectively. None of female patients fulfilled the criteria for sarcopenic obesity, either.

### 3.3. Profiles of NAFLD Patients Associated with Loss of Skeletal Muscle Mass

The details of the eight NAFLD patients with presarcopenia or sarcopenia at entry are listed in Table 3. Except for one male, these patients were not in the criteria for obesity. The body fat percentages in the 7 nonobese patients were higher than 25% in the males and 30% in the females, respectively, indicating that these nonobese patients with muscle volume loss fulfilled the category for “hidden obesity.”

### 3.4. Nutritional and Dietary Factors Associated with SMI

Next, the nutritional and dietary factors associated with the SMI were analyzed at baseline in the male and female NAFLD patients, respectively. As shown in Table 4, the males showed a significantly close correlation between the SMI and the BMI and between the SMI and the IGF-1 level. However, there were no significant differences in the males between SMI and serum ALT, 25-OH vitamin D_3_, zinc, or free testosterone levels. The values of HOMA-IR, FIB4-index, BTR, and BMD in the males were not significantly associated with the SMI, either.

Fifteen of the 24 male NAFLD patients began a diet therapy at baseline. Among the nutritional factors, the estimated total energy and lipid intakes were significantly correlated with SMI in the male patients. Estimated protein intake, leucine intake, and carbohydrate intake were not associated with SMI in the male group.

Among the female patients, the BMI and BMD values were significantly correlated with the SMI at entry (Table 5). Eighteen of 22 female patients received a diet treatment at entry. None of the nutritional factors were revealed to be significantly associated with SMI in the female group.

### 3.5. Changes in Body Compositions and Nutritional Components by the Diet Therapy

Six males and 13 females received a second diet therapy at 6 months after their entry in this study. We determined the changes in these patients' body compositions, nutrients, serum ALT levels, and hand grip strength.

**Figure 1:**
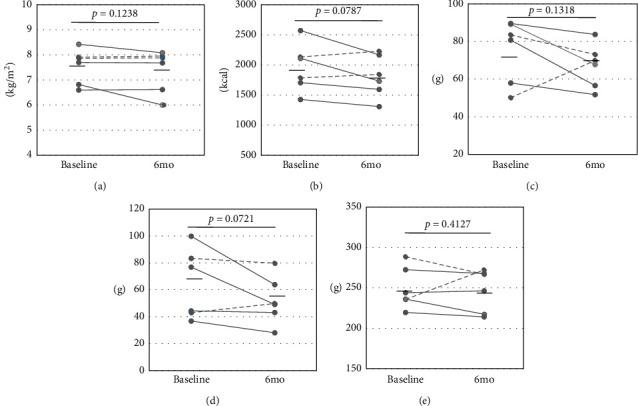
Changes in nutritional components by the diet treatment in the male patients. Horizontal bars represent the average value of each parameter. Broken lines correspond to patients whose total energy intake was increased by the diet treatment. (a) SMI. (b) Total energy intake. (c) Protein intake. (d) Lipid intake. (e) Carbohydrate intake.

**Figure 2:**
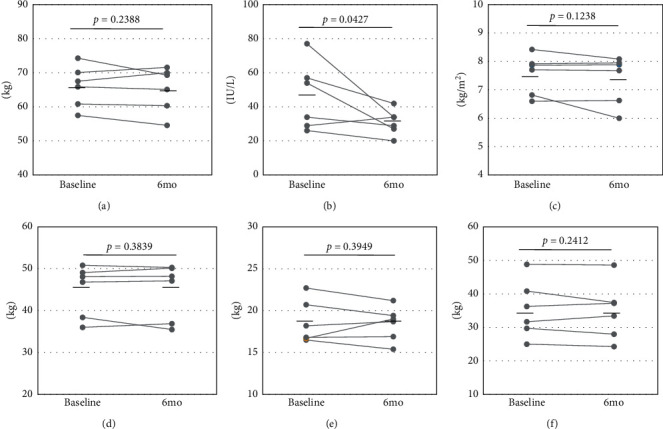
Changes in body compositions by the diet treatment in the male patients. Horizontal bars represent the average value of each parameter. (a) Body weight. (b) ALT. (c) SMI. (d) Skeletal muscle mass. (e) Body fat mass. (f) Hand grip strength.

In the six male patients, the diet therapy tended to decrease the average total energy intake (baseline: 1.957 ± 401 kcal to 6 months: 1.814 ± 349 kcal, *p* = 0.0787, Figure 1). The decrease in energy intake by produced by the diet therapy was responsible for the reduction of protein intake (baseline: 75.2 ± 16.9 g to 6 months: 67.1 ± 11.5 g, *p* = 0.1318) and lipid intake (baseline: 64.0 ± 26.0 g to 6 months: 52.3 ± 17.7 g, *p* = 0.0721), whereas the average carbohydrate intake was approximately equivalent between that at entry and 6 months later (baseline: 249.4 ± 25.8 g to 6 months: 247.4 ± 26.2 g, *p* = 0.4127). The serum ALT level decreased significantly from the baseline to the 6 months measurement (baseline: 46.2 ± 19.9 IU/L to 6 months: 31.0 ± 7.5 IU/L, *p* < 0.05), and the SMI was decreased (baseline: 7.55 ± 0.70 kg/m^2^ to 6 months: 7.37 ± 0.85 kg/m^2^, *p* = 0.1238), although there was no significant difference in SMI between baseline and 6 months later. No significant changes were observed in body weight (baseline: 66.0 ± 6.1 kg to 6 months: 65.2 ± 6.6 kg, *p* = 0.2388), skeletal muscle mass (baseline: 44.9 ± 6.1 kg to 6 months: 44.7 ± 6.7 kg, *p* = 0.3839), body fat mass (baseline: 18.6 ± 2.6 kg to 6 months: 18.4 ± 2.0 kg, *p* = 0.3949), and hand grip strength (baseline: 35.4 ± 8.5 kg to 6 months: 34.8 ± 8.5 kg, *p* = 0.2412) (Figure 2). Four male patients whose energy intake was decreased had lower SMI or approximately equivalent SMI at 6 months, compared with the baseline (Figure 1).

**Figure 3:**
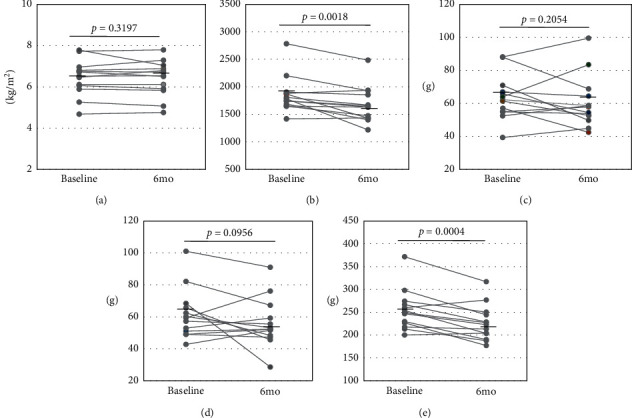
Changes in nutritional components by the diet treatment in the female patients. Horizontal bars represent the average value of each parameter. (a) SMI. (b) Total energy intake. (c) Protein intake. (d) Lipid intake. (e) Carbohydrate intake.

**Figure 4:**
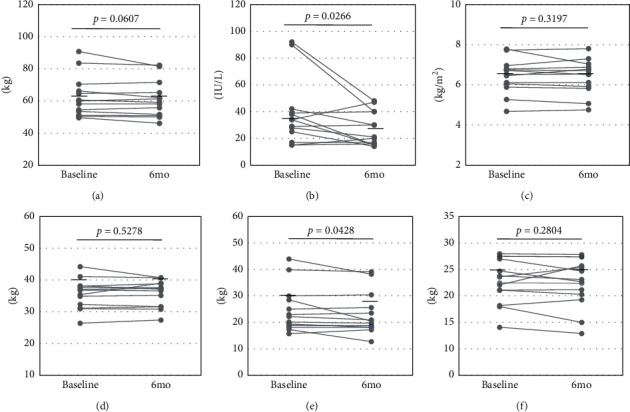
Changes in body compositions by the diet treatment in the female patients. Horizontal bars represent the average value of each parameter. (a) Body weight. (b) ALT. (c) SMI. (d) Skeletal muscle mass. (e) Body fat mass. (f) Hand grip strength.

Among the 13 females, who received the diet therapy, the average total energy decreased significantly from the baseline to 6 months later (baseline: 1.855 ± 331 kcal to 6 months: 1.673 ± 325 kcal, *p* < 0.01, Figure 3). The decrease in total energy intake was derived from lower carbohydrate intake (baseline: 254.4 ± 44.4 g to 6 months: 226.4 ± 38.8 g, *p* < 0.001) and lipid intake (baseline: 61.6 ± 15.6 g to 6 months: 56.0 ± 15.3 g, *p* = 0.0956). The mean protein intake was almost equal between that in the baseline and after 6 months (baseline: 63.6 ± 13.5 g to 6 months: 60.7 ± 15.7 g, *p* = 0.2054). The diet therapy significantly decreased these female patients' serum ALT level (baseline: 38.3 ± 25.0 IU/L to 6 months: 27.2 ± 12.8 IU/L, *p* < 0.05) and body fat mass (baseline: 24.8 ± 8.7 kg to 6 months: 23.3 ± 8.1 kg, *p* < 0.05), and it tended to decrease their body weight (baseline: 62.6 ± 12.7 kg to 6 months: 61.2 ± 11.4 kg, *p* = 0.0607), although no significant changes occurred in the SMI (baseline: 6.44 ± 0.87 kg/m^2^ to 6 months: 6.41 ± 0.86 kg/m^2^, *p* = 0.3191), skeletal muscle mass (baseline: 35.7 ± 4.7 kg to 6 months: 35.7 ± 4.1 kg, *p* = 0.5278), or hand grip strength (baseline: 22.4 ± 4.0 kg to 6 months: 22.1 ± 4.5 kg, *p* = 0.2804) (Figure 4).

## 4. Discussion

Our analyses revealed that sarcopenia and presarcopenia were present in even NAFLD patients. However, the frequencies of presarcopenia and sarcopenia were lower than those in the previous studies [17–19]. One of the reasons for the lower frequency of loss of skeletal muscle mass might be derived from the milder hepatic fibrosis in our patients. There were only two patients among the 46 patients whose livers were assessed as showing advanced hepatic fibrosis. Petta and colleagues reported that sarcopenia was significantly correlated with the severity of hepatic fibrosis in patients with NAFLD [19]. The number of NAFLD patients with more severe hepatic fibrosis was not sufficient. Thus, we may have a bias to evaluate the correlation between the skeletal muscle mass and the severity of hepatic fibrosis in this study.

Contrary to the expectation, none of the NAFLD patients fulfilled the criteria for sarcopenic obesity. Except for one male patient with presarcopenia, all of the patients who met the criteria for sarcopenia and presarcopenia were in the category of nonobese, which is called “hidden obesity.” Our findings established that the SMI was significantly associated with the BMI, implying that loss of skeletal muscle mass was frequently observed in nonobese NAFLD patients rather than obese patients. Our results were quite different from the previous reports that sarcopenic obesity was observed even in patients with NAFLD [18]. Nonobese NAFLD patients tended to have less severe disease at presentation and have a more favorable prognosis than obese patients [40]. To the best of our knowledge, this is the first report of nonobese NAFLD patients being susceptible to loss of skeletal muscle mass. Unfortunately, the clinical definition of “sarcopenic obesity” has not been determined yet. Hence, urgent establishment of the definition is earnestly desired.

Our results demonstrated that the hormonal factors associated with SMI were the IGF-1 level as well as the BMI in the male NAFLD patients. It has been well established that IGF-1 signaling plays an essential role in the formation, maintenance, and regeneration of skeletal muscles via insulin receptor substrate-1 (IRS-1) and mammalian target of rapamycin complex 1 (mTORC1) [5]. It was of interest that a decrease in the serum IGF-1 level was confirmed in an experimental animal model of NAFLD [41]. Therefore, a decrease in IGF-1 production eventually evokes loss of skeletal muscle mass in male [42]. The decrease in growth hormone (GH) release is likely to cause the decline of IGF-1 production, but it does not affect muscle strength [43]. Unfortunately, we did not determine serum GH level in each patient. Further examination will be required to clarify that.

In contrast, our findings elucidated that the factors linked to the SMI were the BMI and the BMD in female patients with NAFLD. Yilmaz noted that concurrent osteoporosis was frequently observed in patients with NAFLD [44]. The concept of biochemical communications between bones and muscles has been proposed. Several types of growth factors, myokines, and osteokines are thought to affect both bones and muscles. Therefore, sarcopenia may develop more severely in proportion to the severity of osteoporosis [45]. It has been well documented that the deterioration of BMD frequently causes sarcopenia in patients with NAFLD [46]. It is conceivable that vitamin D_3_ deficiency was commonly observed in our NAFLD patients [47].

Estrogen is also a crucial factor for skeletal growth and the maintenance of skeletal integrity in women. A decrease in estrogen secretion in females is likely to ameliorate the skeleton's responsiveness to exercise more than in males, leading to loss of skeletal muscle mass [42]. These results described above may indicate that the hormonal factors that contribute to the regulation of the skeletal muscle mass were quite distinct between male and female NAFLD patients.

The results of our analyses did not demonstrate that the patients' muscle mass was associated with insulin resistance, the free testosterone, vitamin D_3_, or zinc levels. Lee and colleagues previously revealed that loss of skeletal muscle mass was unrelated to insulin resistance and correlated only with the severity of hepatic fibrosis in such patients [17], which is consistent with our present findings. Higher frequency of vitamin D_3_ deficiency in female patients may account for higher frequency of skeletal muscle mass loss than in male patients in this study. Direct evidence that zinc deficiency evoked loss of skeletal muscle mass was not obtained. An indirect correlation may thus be present between the zinc level and skeletal muscle mass. We did not determine the patients' serum selenium level, total testosterone, or estradiol concentrations. These should be examined in a future study.

Our results demonstrated that the dietary factors that contribute to the regulation of the skeletal muscle mass in the male patients were the total energy intake and the lipid intake. Hayashi and colleagues revealed that insufficient energy intake was significantly associated with sarcopenia in patients with compensated liver cirrhosis [48], supporting our results in the present study. However, no significant dietary factors associated with SMI were revealed in our female NAFLD patients. These results may suggest that male patients are likely to affect the dietary factors more strongly than female patients. Surprisingly, we did not obtain evidence that the protein intake was associated with the skeletal muscle mass, which was the opposite of a previous study's results [49]. It has been well recognized that leucine directly activates mTORC1 that stimulates protein synthesis and decreases autophagy [50]. We expected that the leucine intake was associated with the skeletal muscle mass in the enrolled patients. However, the leucine intake did not particularly contribute any regulation of the skeletal muscle mass in such patients at all.

The effects of the dietary intervention on body composition were also different between our male and female groups. The total energy intake was decreased by way of a decline in their protein and lipid intakes due to the diet treatment in male patients. Consequently, the SMI value was decreased 6 months later, although the improvement of fatty liver was observed in such patients. This finding may support our finding that the muscle mass in the male patients was associated with the total energy intake and the lipid intake. In the female patients, a decreased total energy intake was accomplished by the diet intervention via reduction in their lipid intake and carbohydrate intake. The change in their protein intake was not observed in such patients. Then, fatty liver was improved and the SMI was maintained in the female patients. Taking these results into consideration, it appears that sufficient protein intake is necessary to maintain the muscle mass in NAFLD patients, although our study confirmed that the protein intake was not associated with the skeletal muscle mass in such patients. Further examinations are necessary to clarify this discrepancy. Moreover, the effects of diet treatment for NAFLD appeared to be entirely distinct between male and female patients.

There are several limitations to address. The sample size was small (*n* = 46) in our study, although many useful results were obtained. A larger-scale cohort study is required for confirmation of our results. Second, we did not perform histological examinations of the livers of the enrolled patients. We were unable to investigate the correlations between skeletal muscle mass and hepatic fibrosis or hepatic steatosis in the enrolled patients. Third, most of the enrolled patients were predicted to show mild hepatic fibrosis, because most of FIB-4 index values in the enrolled patients were under 2.67. We therefore did not sufficiently evaluate the skeletal muscle mass in NAFLD patients with advanced hepatic fibrosis in this study. Fourth, a qualitative evaluation of skeletal muscle was not performed in this study. The severity of myosteatosis, characterized by excessive fat deposition into the skeletal muscle, was not evaluated at all. We examined the skeletal muscle mass alone in this study. Fifth, a period for the dietary assessments was only three days. In addition, the dietary assessments were based largely on the patients' declarations. Thus, we have limitations to more detailed and objective evaluation for the dietary assessments. Finally, we did not evaluate the effect of the physical activity in each patient, although we confirmed the frequency of the physical training in a week. Then, the data obtained from this study might have a bias in the nutritional and dietary assessment.

## 5. Conclusions

Our results reveal that loss of skeletal muscle was frequently present in nonobese patients with NAFLD and that the frequency of sarcopenic obesity appears to be low among NAFLD patients. The nutritional and dietary factors associated with skeletal muscle index were distinct between the male and female NAFLD patients. The dietary intervention was effective for fatty liver but was harmful for skeletal muscle mass in some presarcopenic or sarcopenic patients. Therefore, the skeletal muscle mass as well as the body weight and liver function should be monitored during dietary intervention for NAFLD patients.

## Figures and Tables

**Table 1:** Clinical characteristics of the enrolled patients.

Parameter	Male (*n* = 24)	Female (*n* = 22)	*p* value
Age (years)	50.4 ± 14.0	56.3 ± 9.4	0.1037
BMI (kg/m^2^)	26.2 ± 3.0	26.0 ± 3.7	0.7585
SMI (kg/m^2^)	7.80 ± 0.72	6.44 ± 0.82	<0.0001
Hand grip strength (kg)	37.7 ± 6.5	23.2 ± 3.8	<0.0001
BMD (g/cm^2^)	1.26 ± 0.31	1.03 ± 0.17	0.1184
ALT (IU/L)	46.0 ± 31.0	31.9 ± 21.6	0.0765
FIB-4 index	1.10 ± 0.50	1.40 ± 0.77	0.2060
LDL-C (mg/dl)	123.9 ± 26.9	138.2 ± 37.9	0.1263
TG (mg/dl)	142.1 ± 84.8	148.2 ± 81.0	0.6521
HbA1c (%)	5.73 ± 0.42	5.88 ± 0.53	0.6139
HOMA-IR	2.04 ± 1.04 (*n* = 10)	2.89 ± 1.70 (*n* = 9)	0.2701
BTR	7.48 ± 1.34 (*n* = 23)	6.66 ± 1.12	0.0495
25-OH-vitamin D3 (ng/ml)	18.4 ± 6.1 (*n* = 23)	16.1 ± 4.1	0.3622
Zinc (*μ*g/dl)	76.7 ± 12.9 (*n* = 23)	72.8 ± 11.3	0.3754
Free testosterone (pg/ml)	9.3 ± 3.2	ND	ND
Concurrent T2DM	5 (17%)	6 (27%)	0.4336
Concurrent dyslipidemia	10 (42%)	13 (59%)	0.1881
Concurrent hypertension	4 (17%)	3 (14%)	0.551

ND, not determined.

**Table 2:** Frequencies of presarcopenia, sarcopenia, and sarcopenic obesity in the male and female NAFLD patients.

Criteria	Male (*n* = 24)	Female (*n* = 22)	*p* value
Presarcopenia	3 (13%)	4 (18%)	0.449
Sarcopenia	0 (0%)	1 (5%)	0.4783
Sarcopenic obesity	0 (0%)	0 (0%)	0.9999

**Table 3:** Profile of the NAFLD patients with loss of skeletal muscle mass.

Gender	Age	Criteria	SMI	Hand grip strength	BMI	Body fat (%)
Female	75	Sarcopenia	4.68	rt: 15.2 kg	22.7	44.1
				lt: 13.5 kg		

Female	52	Presarcopenia	5.45	rt: 19.2 kg	20.3	31.7
				lt: 19.1 kg		

Female	54	Presarcopenia	5.58	rt: 26.1 kg	19.5	28.9
				lt: 21.5 kg		

Female	62	Presarcopenia	5.27	rt: 20.0 kg	21.1	35.5
				lt: 22.1 kg		

Female	75	Presarcopenia	5.73	rt: 20.5 kg	23.8	35.2
				lt: 23.2 kg		

Male	67	Presarcopenia	6.33	rt: 31.6 kg	23.6	32.6
				lt: 28.6 kg		

Male	34	Presarcopenia	6.99	rt: 37.9 kg	24.4	32.6
				lt: 34.0 kg		

Male	63	Presarcopenia	6.6	rt: 26.2 kg	26.1	37.4
				lt: 26.5 kg		

rt, right; lt, left.

**Table 4:** Nutritional and dietary factors associated with SMI in male patients at baseline.

Parameter	*r*	*p* value
ALT (*n* = 24)	0.0596	0.7749
IGF-1 (*n* = 19)	0.5426	0.0212
HOMA-IR (*n* = 10)	−0.0182	0.9565
25-OH-vitamin D3 (*n* = 23)	0.1269	0.5518
Zinc (*n* = 23)	−0.2825	0.1851
BTR (*n* = 23)	0.083	0.697
FIB-4 index (*n* = 24)	−0.0509	0.8072
BMD (*n* = 7)	0.6785	0.0965
Free testosterone (*n* = 13)	0.2418	0.4023
BMI (*n* = 24)	0.657	0.0016
Total energy intake (*n* = 15)	0.6429	0.0162
Protein intake (*n* = 15)	0.1036	0.6984
Leucine intake (*n* = 15)	0.1464	0.5838
Lipid intake (*n* = 15)	0.5429	0.0422
Carbohydrate intake (*n* = 15)	0.0643	0.8099

**Table 5:** Nutritional and dietary factors associated with SMI in female patients at baseline.

Parameter	*r*	*p* value
ALT (*n* = 22)	0.1352	0.5355
IGF-1 (*n* = 18)	0.1839	0.4483
HOMA-IR (*n* = 9)	0.0333	0.9249
25-OH-vitamin D3 (*n* = 22)	0.0877	0.6879
Zinc (*n* = 22)	0.0724	0.7401
BTR (*n* = 22)	−0.1621	0.4577
FIB-4 index (*n* = 22)	−0.3845	0.078
BMD (*n* = 10)	0.697	0.0366
BMI (*n* = 22)	0.8944	<0.0001
Total energy intake (*n* = 18)	−0.1847	0.4463
Protein intake (*n* = 18)	−0.129	0.5948
Leucine intake (*n* = 18)	−0.2817	0.2454
Lipid intake (*n* = 18)	−0.0258	0.9152
Carbohydrate intake (*n* = 18)	−0.2425	0.3173

## Data Availability

Patients' data included within this article are also available from the corresponding author upon request.
